# Binding of two DNA molecules by type II topoisomerases for decatenation

**DOI:** 10.1093/nar/gks843

**Published:** 2012-09-18

**Authors:** Rupesh Kumar, Jane E. Riley, Damian Parry, Andrew D. Bates, Valakunja Nagaraja

**Affiliations:** ^1^Department of Microbiology and Cell Biology, Indian Institute of Science, Bangalore 560012, India, ^2^Institute of Integrative Biology, University of Liverpool, Biosciences Building, Liverpool L69 7ZB, UK and ^3^Jawaharlal Nehru Centre for Advanced Scientific Research, Bangalore 560064, India

## Abstract

Topoisomerases (topos) maintain DNA topology and influence DNA transaction processes by catalysing relaxation, supercoiling and decatenation reactions. In the cellular milieu, division of labour between different topos ensures topological homeostasis and control of central processes. In *Escherichia coli,* DNA gyrase is the principal enzyme that carries out negative supercoiling, while topo IV catalyses decatenation, relaxation and unknotting. DNA gyrase apparently has the daunting task of undertaking both the enzyme functions in mycobacteria, where topo IV is absent. We have shown previously that mycobacterial DNA gyrase is an efficient decatenase. Here, we demonstrate that the strong decatenation property of the enzyme is due to its ability to capture two DNA segments in *trans*. Topo IV, a strong dedicated decatenase of *E. coli,* also captures two distinct DNA molecules in a similar manner. In contrast, *E. coli* DNA gyrase, which is a poor decatenase, does not appear to be able to hold two different DNA molecules in a stable complex. The binding of a second DNA molecule to GyrB/ParE is inhibited by ATP and the non-hydrolysable analogue, AMPPNP, and by the substitution of a prominent positively charged residue in the GyrB N-terminal cavity, suggesting that this binding represents a potential T-segment positioned in the cavity. Thus, after the GyrA/ParC mediated initial DNA capture, GyrB/ParE would bind efficiently to a second DNA in *trans* to form a T-segment prior to nucleotide binding and closure of the gate during decatenation.

## INTRODUCTION

DNA topoisomerases (topos) facilitate many DNA transaction processes by altering the topology of DNA molecules, and are essential for cell survival (1–5). Based on the mechanism used for inter-conversion of different topoisomers, the enzymes have been classified as type I or type II (6), and further into IIA and IIB, on the basis of structural considerations (7). Type IIA topos, which occur in all eubacteria and eukaryotes, bind and catalyse the double-strand cleavage of a DNA segment, termed the gate (G) segment, followed by the ATP-dependent transfer of another duplex DNA, the transfer (T) segment, through the break and subsequent religation of the cleaved segment. Type IIA topos are the target of a number of classes of cytotoxic compounds, which act to stabilize the transient double-stranded break, leading to DNA breakage and cell death. These include anti-tumour agents targeting the human enzymes (8,9) and anti-bacterial compounds, most notably the quinolones and fluoroquinolones (10,11).

Using this double-strand passage mechanism, type IIA topos can relax both positive and negative supercoiling, with a linking number change of ±2 per cycle (6,7,12), and can also catalyse decatenation of the catenated intermediates formed during replication (5,13), and can unknot DNA (14). All cells require at least one type II topo, but bacteria contain a specialized type IIA enzyme, DNA gyrase, that can introduce negative supercoils using the free energy of ATP hydrolysis (15). In *Escherichia coli*, and many other bacteria, while gyrase is responsible for the introduction of negative supercoils (15), as well as the removal of positive supercoils ahead of replication and transcription (4,16), the topo IV paralogue acts to separate the catenated daughter replication products (17,18), relax DNA (18,19) and remove potentially lethal knots (14,20,21). Thus, topo IV preferentially carries out inter-molecular strand passage, resulting in decatenation, while DNA gyrase primarily catalyses intra-molecular strand passage, leading to the accumulation of negative supercoils (22). This primary difference in the reactions of the two enzymes appears to be conferred by a different mode of selection of the substrate T-segment. In prokaryotes, these enzymes are heterotetramers of two subunits, one containing the active site tyrosine residue for the cleavage and religation of the G-segment (GyrA in gyrase, ParC in topo IV), the other (GyrB, ParE), the ATPase active site (6,23). The selection of a particular substrate by topo IV (24) or gyrase (25) depends upon the geometry of the DNA, which is substantially determined by the carboxy terminal domain (CTD) of GyrA/ParC (24–26). Crystal structures of the CTD of ParC (27) and GyrA (28) reveal the basis of this differential preference. The GyrA CTD adopts a β-pinwheel fold, responsible for wrapping the DNA at either side of the G-segment region. Gyrases thus bind ∼130 bp of DNA, and the wrapping of DNA around the GyrA CTD serves to position a contiguous T-segment in a cavity between the GyrB N-terminal domains (29–31) for ‘capture’ by dimerization of those domains on ATP binding (32–35), leading to strand-passage and negative supercoiling. In contrast, the ParC CTD contains a broken β-pinwheel fold due to the absence of the ‘GyrA-box’ motif (27). This reduces the curvature of the proposed DNA-binding region, resulting in less efficient DNA bending by ParC (24,27). Topo IV thus does not facilitate the selection of a T-segment in *cis*, and is more likely to capture a T-segment from another DNA molecule, resulting in decatenation, and accordingly, the enzyme cannot supercoil DNA, but is a strong decatenase. In contrast, gyrase facilitates the introduction of negative supercoils and is a very poor decatenase (22).

Unlike in *E. coli,* where a division of labour ensures supercoiling and decatenation are performed by different enzymes, in organisms of the genus *Mycobacteria*, a single type II enzyme appears to suffice. None of the mycobacterial species whose genome has so far been sequenced possesses a topo IV homologue; DNA gyrase is the sole type II topo in this genus with the exception of a second type II enzyme with unusual properties in *Mycobacterium smegmatis* (36). Several other eubacteria also lack topo IV (37). Thus, in these organisms, either DNA gyrase carries out the additional task of separating catenated daughter chromosomes, or an alternative mechanism must have evolved to perform this essential task (38). Since GyrA and GyrB subunits from the two mycobacterial species show only ∼40–44% identity to *E. coli* gyrase subunits, they are likely to exhibit distinct properties. Accordingly, we found that DNA gyrase from *M. smegmatis* is an efficient decatenase (39). *Mycobacterium tuberculosis* DNA gyrase has also been shown to be a potent decatenase [(40); our unpublished results], suggesting that gyrase could be carrying out this dual role *in vivo* in these organisms.

In the present study, we have investigated what makes the mycobacterial DNA gyrase able to perform efficient decatenation, a task performed efficiently by topo IV, which is functionally different from a typical DNA gyrase. We present evidence for the efficient binding of two separate DNA molecules by *M. smegmatis* gyrase, a property that is shared with *E. coli* topo IV, but not with the ‘typical’ *E. coli* gyrase. The binding site for the second DNA in both the enzymes lies within their ATPase subunits. The data are consistent with the idea that this second DNA corresponds to the captured T-segment.

## MATERIALS AND METHODS

### Enzymes and chemicals

Oligonucleotides used in this study are listed in Supplementary Table S1. DNA I (40 bp) contains the strong gyrase site sequence mapped for mycobacterial gyrase (39). DNA II (72 bp) contains 16 additional residues flanking each side of the DNA I. DNA III (143 bp) is PCR amplified using 5′-end-labelled pUC18 forward and unlabelled reverse primers, from pUC18 containing DNA I cloned in the multiple cloning site. The 240-bp DNA was PCR amplified from the pBR322 strong gyrase site sequence (29). Oligonucleotides (MWG and Sigma Aldrich) were gel purified and end labelled using T4 polynucleotide kinase (NEB) and γ-^32^P ATP (6000 Ci/mmol). Labelled oligonucleotides were further purified using a 1 ml G-25 sephadex (Sigma Aldrich) spin column and annealed to their respective complementary oligonucleotides. Ampicillin, kanamycin, IPTG and acetamide were from Sigma Aldrich. Purification of *E. coli* DNA gyrase, *M. smegmatis* DNA gyrase and *E. coli* topo IV subunits were carried out as described previously (19,39,41).

The R321A mutant of *M. smegmatis* GyrB was generated using the megaprimer inverse PCR method (42). The mutation was confirmed by sequencing. The oligonucleotides used for mutagenesis are listed in Supplementary Table S1. Mutant GyrB was purified following the protocol used for wild-type mycobacterial GyrB. A plasmid based on pET17b (Novagen) was constructed for the expression of residues 1–391 of *E. coli* ParE (pJR1, expressing ParE43). The R284Q mutation was constructed using Quikchange mutagenesis (Strategene) to replace a CGT with a CAG codon, and confirmed by sequencing. The proteins were expressed in *E. coli* strain B834 (DE3) pLysE (Novagen), by induction with IPTG (0.4 mM) and subsequently purified to homogeneity by heparin–Sepharose and Mono-Q Sepharose (GE Healthcare) chromatography at pH 7.5. The proteins were stored in 50 mM Tris–HCl (pH 7.5), 100 mM KCl, 5 mM DTT, 1 mM EDTA and 10% (w/v) glycerol. Protein concentrations were determined using Bradford’s method (43).

### Electrophoretic mobility shift assay

Different concentrations of the protein (as indicated in the Figure legends) were incubated with 1 nM of end-labelled double-stranded DNA fragment (sizes as indicated in the Figure legends) in supercoiling buffer (35 mM Tris–HCl pH 7.5, 1 mM spermidine, 25 mM potassium glutamate, tRNA 90 µg/ml, 1.4 mM ATP and BSA 50 µg/ml) at room temperature for 15 min. The complexes were resolved on 5% native PAGE (0.5× TBE buffer). Gels were dried and exposed to phosphor imager film and imaged by a Fujifilm phosphor imager (FLA 15 000).

### Biotin pulldown assay

For biotin pulldown assays, 72-bp biotinylated oligonucleotide was annealed to its complimentary oligonucleotide (^32^P-labelled). The gyrase holoenzyme or subunits were incubated first with either biotinylated DNA (72 bp) or non-biotinylated 240-bp DNA in supercoiling reaction buffer at 25°C for 10 min followed by the addition of the other DNA. After 5 min, streptavidin–sepharose beads were added to each of the reaction and allowed to bind to biotinylated DNA for 15 min by continuous mixing. The beads were pelleted by centrifugation and washed using supercoiling reaction buffer. The pellet and the supernatant fractions were analysed on a 7% urea PAGE. The gels were dried and exposed to phosphor imager film and imaged by Fujifilm phosphor imager FLA 15 000.

### SPR-binding measurements

Surface plasmon resonance (SPR)-binding measurements of ParE43 and the R284Q mutant to DNA were carried out with a BIAcore X system (BIAcore). A 140-bp DNA fragment, amplified from plasmid pBR322, and biotinylated at one 5′-end, was bound to a streptavidin sensor chip. Measurements were carried out in 33 mM Tris–HCl (pH 8.0), 5 mM MgCl_2_, 3 mM DTT at a flow rate of 30 µl min^−^^1^ at 25°C. Where indicated, the protein was pre-incubated with ADPNP (0.5 mM) for 30 min at 37°C. The data were analysed using BIAevaluation software (BIAcore).

### Crosslinking experiments

DNA gyrase or individual subunits were incubated with 5′-end-labelled 72-bp DNA II (Supplementary Table S1) in the presence or absence of ATP (as indicated) in the supercoiling buffer for 30 min on ice after which crosslinking solution (37% formaldehyde and Methanol; 9:1) was added to a final concentration of 0.7%. Crosslinking reactions were continued for 30 min at 37°C (44). The products were separated by 8% SDS–PAGE and visualized using a phosphor imager.

Protein crosslinking assays were carried out using glutaraldehyde. DNA gyrase subunits were mixed in a 1:1 ratio and incubated in supercoiling reaction buffer on ice for 30 min followed by the addition of 1 µl of 0.2% of glutaraldehyde. Reactions were shifted to 37°C for 30 min, and terminated by the addition of SDS to a final concentration of 0.1%. Crosslinked products were resolved on 6% SDS–PAGE (30:1 acrylamide and bisacrylamide ratio) and visualized by silver staining.

### ATPase assay

The ATPase reactions were carried out in a buffer containing 40 mM Tris–HCl, pH 7.5, 5 mM MgCl_2_, 10 mM DTT, 1 mM spermidine, 20 mM KCl, 1.4 mM ATP and 0.02 µCi of [γ-^32^P] ATP (6000 Ci mmol^−^^1^). To assess the intrinsic ATPase activity, 100 nM of GyrB/ParE subunits and *M. smegmatis* DNA gyrase/ topo IV holoenzyme were used. For DNA-dependent stimulation of ATPase activity, 100 or 400 nM of 72-bp DNA was added as indicated. The assays were carried out at 37°C for 60 min followed by chloroform extraction. The aqueous layer (1 µl) was resolved on polyethyleneimine-cellulose thin layer chromatography using 1.2 M LiCl, and 0.1 mM EDTA as mobile phase. The spots of ATP and *P*_i_ were visualized using a phosphor imager and quantitated using Image gauge software. ATPase assays of ParE43 and the R284Q mutant were carried out using a pyruvate kinase–lactate dehydrogenase coupled system in 50 mM Tris–HCl, 5 mM DTT, 100 mM KCl, 2 mM MgCl_2_ and 2 mM ATP at 37°C, including concentrations of linear pBR322 DNA (4361 bp) as described (45).

### Supercoiling and decatenation

Three-hundred nanograms of pUC18 DNA relaxed with *E. coli* Topo I was used as a substrate for supercoiling reactions with 25 nM of either *E. coli* or *M. smegmatis* DNA gyrase. The reactions were carried out in supercoiling buffer at 37°C for 30 min and terminated by the addition of 0.1% SDS (final concentration) followed by proteinase K (50 µg/ml) treatment for 1 h at 37°C. The topoisomers were resolved on 1.2% agarose gels.

Decatenation assays were carried out using *Leishmania donovani* kDNA. An amount of 25 nM of the enzyme and 300 ng of kDNA were used for each reaction. The assays were carried out as described earlier (39) and terminated by the addition of gel-loading dye (0.6% SDS, 0.25% bromophenol blue, 0.25% xylene cyanol, 1 mM EDTA and 5% glycerol) followed by heat inactivation at 75°C for 10 min. Released minicircles were resolved on 1.2% agarose gels stained with ethidium bromide. The gels were visualized in a Biorad gel documentation system.

### Filter-binding assay

Filter-binding assays were carried out using a 12-well Millipore filtration apparatus and 0.45-µm pore-sized nitrocellulose filters (Millipore). Reaction mixtures (20 µl) were as described for electrophoretic mobility shift assay (EMSA). An amount of 20 nM of GyrB (*M. smegmatis* and *E. coli*) and ParE (*E. coli*) were used for binding to 72-bp DNA. After incubating for 10 min on ice, the assay mixtures were spotted on nitrocellulose filters, loaded on the filtration apparatus and pre-washed with a wash buffer (1 ml) containing 35 mM Tris–HCl, 10% (v/v) glycerol, 0.1 mM EDTA, 50 mM NaCl and 2 mM 2-mercaptoethanol. After 5 min, each filter was washed twice with 1 ml of wash buffer to remove any non-specifically bound DNA. The filters were dried and counts retained were measured by scintillation counter.

## RESULTS

### Experimental design

The essential difference between the two major reactions catalysed by type II topos is the mode of T-segment capture. For a supercoiling reaction involving intra-molecular strand passage, gyrase has to capture the T-segment in *cis*. In contrast, topo IV has to capture a separate DNA in *trans* for inter-molecular strand passage during the decatenation reaction. We therefore hypothesize that a good decatenase, such as *M. smegmatis* DNA gyrase, must be able to bind efficiently to a second DNA segment, in addition to the G-segment. Thus, we compared the DNA-binding properties of *M. smegmatis* and *E. coli* gyrase to DNA fragments of varying sizes, using EMSA and DNA crosslinking assays. *E. coli* topo IV, a very strong decatenase, was also used to monitor the DNA binding in control experiments.

### *Mycobacterium smegmatis* DNA gyrase can bind two DNA molecules

**Figure 1.**
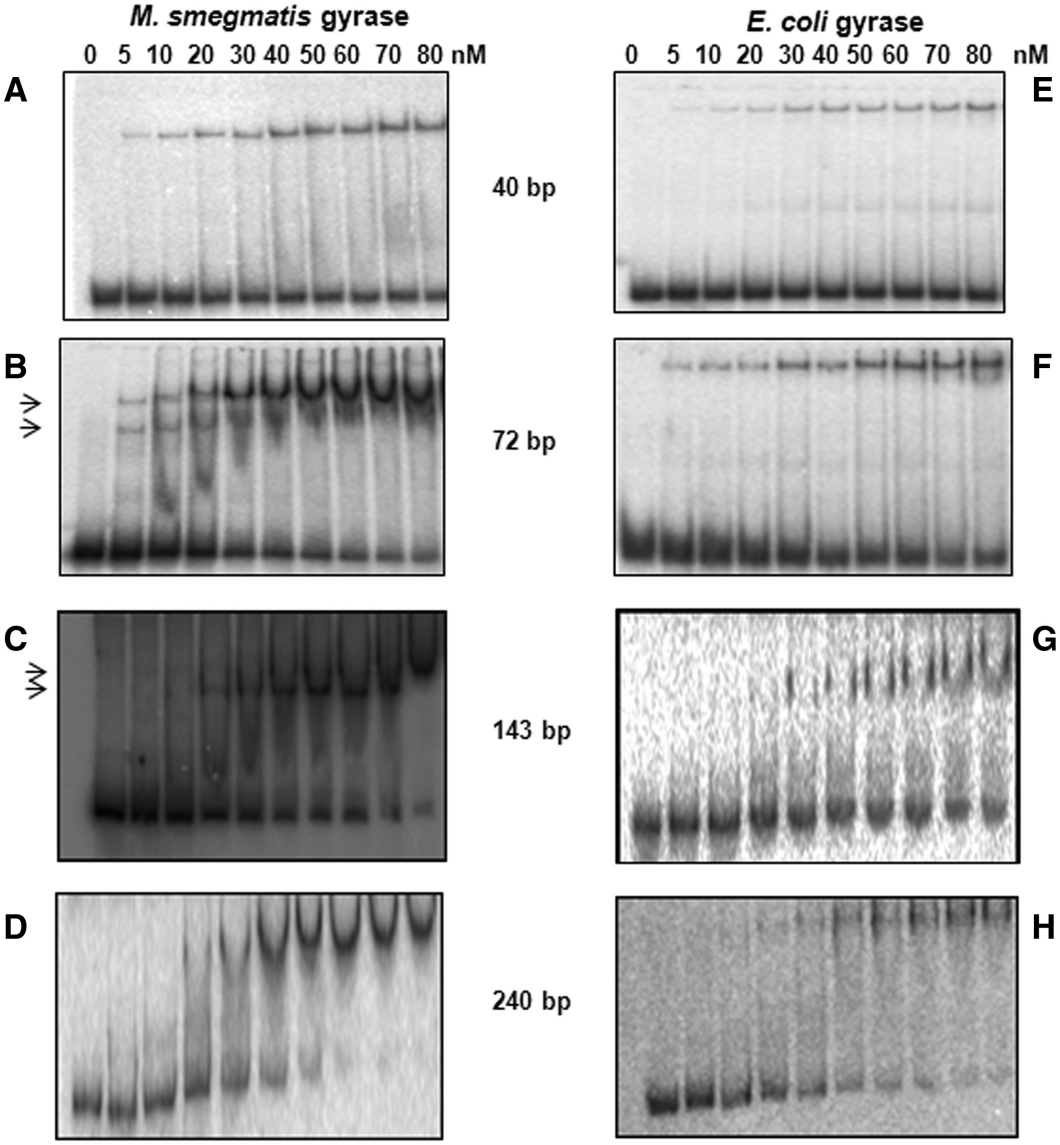
DNA-binding properties of *M. smegmatis* and *E. coli* DNA gyrase. EMSA carried out with increasing concentrations of MsGyr *(M. smegmatis)* or EcGyr (*E. coli*) gyrase with radioactively labelled DNA molecules of different lengths. DNA binding with MsGyr (**A–D**) and EcGyr (**E–H**). The reactions were carried out at 25°C and then resolved on 5% native polyacrylamide gels using 0.5× TBE buffer and visualized using a phosphorimager. The arrows indicate two DNA-bound complexes in (B) and (C). Details of the reactions are given in ‘Materials and Methods’ section. For sequences of the DNA molecules refer to Supplementary Table S1.

We monitored binding of different length DNA substrates by EMSA, using both *M. smegmatis* and *E. coli* gyrase (Figure 1 and Supplementary Figure S1). With the shortest DNA (40 bp), formation of only one complex was seen with both the gyrases irrespective of the enzyme concentration (Figure 1A and E). In contrast, two complexes were observed using a 72-bp DNA, at lower concentrations of the mycobacterial gyrase; increasing the enzyme concentration led to the formation of a single slower mobility complex (Figure 1B), suggesting the possibility of binding two DNA molecules independently to the same enzyme. Similar results were obtained with a 143-bp DNA; a faster moving species was converted into a supershifted complex at the highest enzyme concentration (Figure 1C). In contrast, when we used a much larger DNA (240 bp), only one complex was formed (Figure 1D). Equivalent assays carried out with *E. coli* gyrase revealed only one complex with all the DNA molecules (40–240 bp) used (Figure 1E–H).

**Figure 2.**
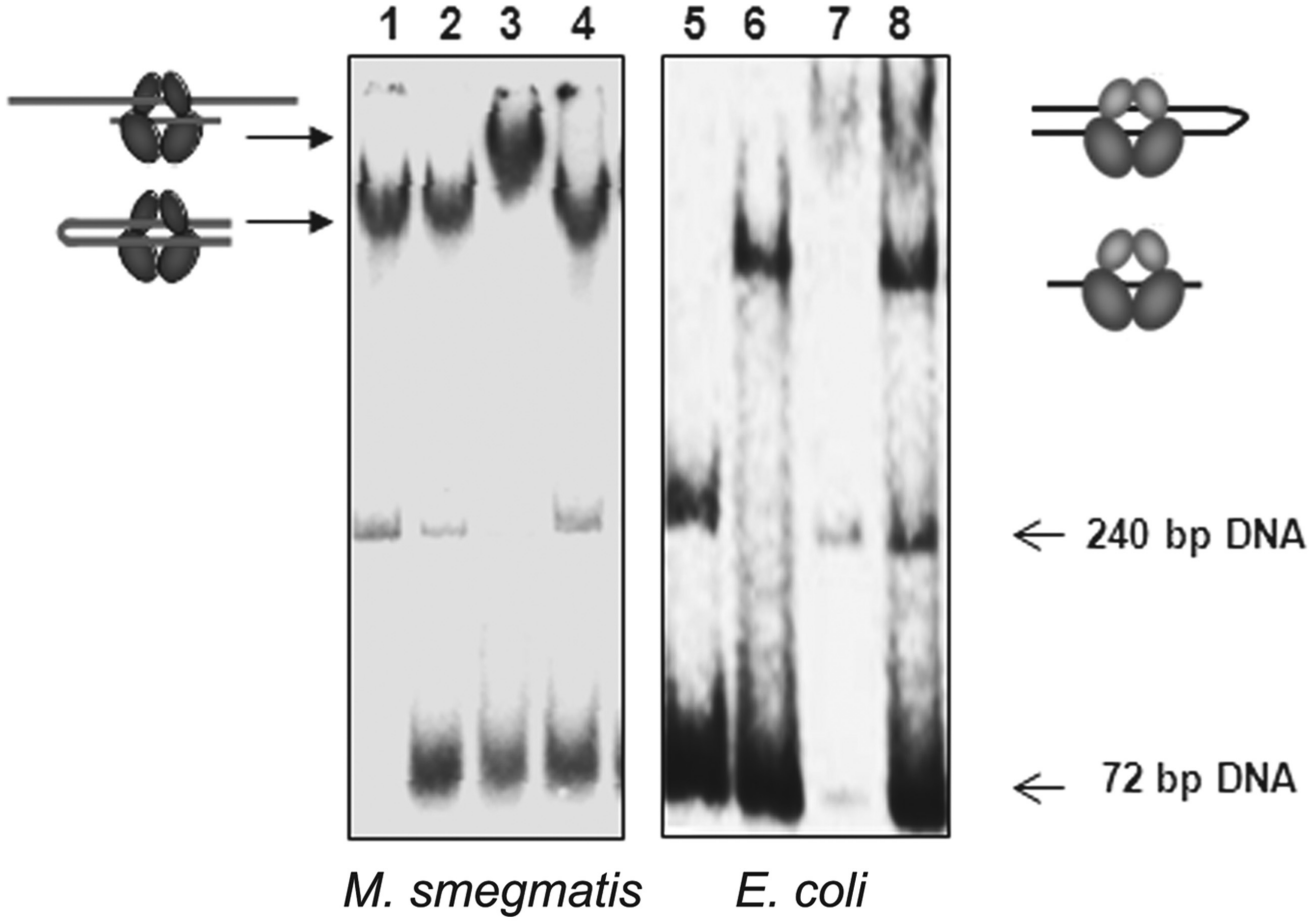
EMSA of *M. smegmatis* and *E. coli* gyrase with 72- and 240-bp DNA. DNA gyrase (50 nM) was incubated with DNA and 2 µg/ml MFX at 37°C for 10 min and then shifted back to ice before adding the second DNA. The 72- and 240-bp DNA used were the same as in Figure 1. Lane 1—MsGyr + 240 bp, Lane 2—MsGyr + 240 bp→72 bp (where the arrow-head indicates order of addition), Lane 3—MsGyr + 72 bp+ MFX→240 bp, Lane 4—MsGyr + 240 bp+ MFX→72 bp, Lane 5—Free 72- and 240-bp DNA, Lane 6-EcGyr + 72 bp, Lane 7-EcGyr + 240 bp, Lane 8—EcGyr + 72 bp+ MFX→240 bp. The complexes were resolved on a 5% native polyacrylamide gel and visualized using a phosphor imager. The pictograms indicate the likely mode of binding of the DNAs to the gyrase holoenzymes.

To further assess the properties of the two DNA-binding sites, the ability of the quinolone drugs to stabilize a covalent complex with a cleaved G-segment was used. Stabilization of binding using a quinolone should ensure that a DNA fragment is bound as a G-segment, allowing binding of additional DNA fragments to be investigated. Moxifloxacin (MFX) was used in these experiments, since it is a better inhibitor of mycobacterial gyrase than other fluoroquinolones (39). As seen previously, gyrase can form a well-defined complex with a 240-bp DNA (Figure 2, Lane 1), which is unaffected by the subsequent addition of 72-bp DNA, with or without pre-treatment with MFX (Lane 2, Lane 4). However, when the 72-bp DNA and the *M. smegmatis* enzyme were initially trapped in a covalent complex using MFX, followed by the addition of 240-bp DNA, a supershifted complex was formed (Lane 3). In contrast, *E. coli* gyrase did not show any evidence of a supershifted complex containing both DNAs (Lanes 6–8).

Taken together, these experiments suggest that *M. smegmatis* DNA gyrase can bind two separate DNA segments, but binding of a long fragment initially as a G-segment inhibits the subsequent binding of a second fragment, presumably because the 240-bp fragment is able to form a proficient DNA wrap, and additionally fill the second DNA-binding site, potentially the site of T-segment binding.

To confirm that mycobacterial gyrase has the ability to bind two different DNA molecules efficiently, biotin pulldown assays were also carried out using biotinylated 72-bp and untagged 240-bp fragments (Supplementary Figure S2A); the experimental design is described in the Supplementary Figure S2B and in ‘Materials and Methods’ section. When the 72-bp DNA was added to mycobacterial gyrase first, followed by the 240-bp, both the DNAs were found predominantly in the pellet fraction, suggesting that the enzyme had bound both DNAs. In contrast, when the order of DNA addition was reversed i.e. with the initial binding of a larger segment, only shorter biotinylated DNA was predominantly found in the pellet (Supplementary Figure S2C). Using *E. coli* gyrase, co-precipitation of the two fragments was much less efficient. These results are consistent with the data from mobility shift experiments described above. Only with mycobacterial gyrase and a short G-segment is efficient binding of the second DNA molecule seen.

### *Mycobacterium smegmatis* GyrB binds DNA

**Figure 3.**
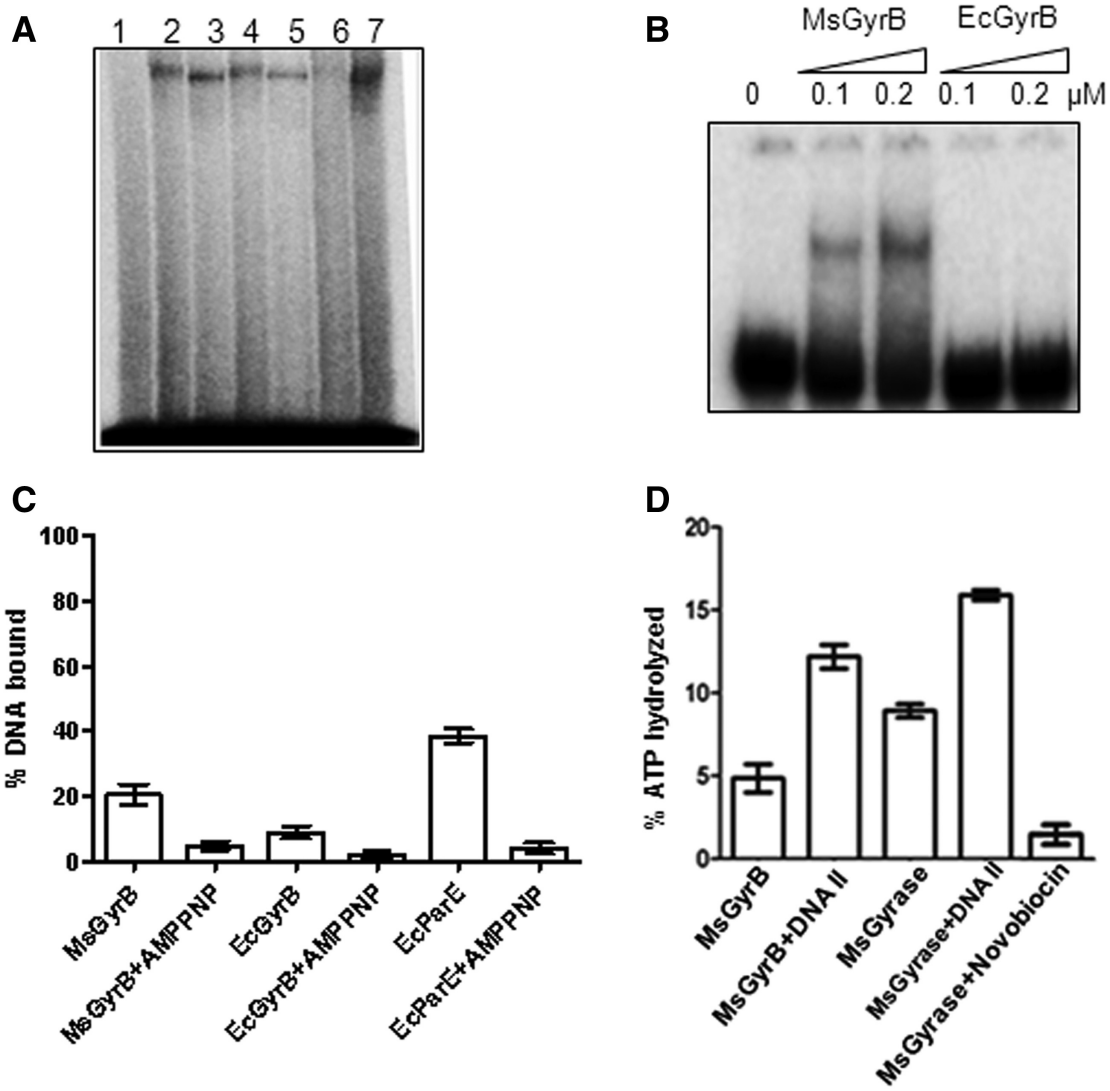
DNA binding by GyrB. (**A**) Crosslinking of DNA gyrase holoenzymes and individual subunits from *M. smegmatis* and *E. coli* with 72-bp DNA. An amount of 100 nM of GyrA subunit and 1 µM of each of the GyrB subunits were used for individual subunit crosslinking. The holoenzymes were reconstituted by mixing the 100 nM of GyrA subunit with 200 nM of the respective GyrB subunits. DNA crosslinking with gyrase and its individual subunits was carried out as described in ‘Materials and Methods’ section and the products were resolved on an 8% SDS–PAGE. Lane 1—DNA only, Lane 2—EcGyr, Lane 3—MsGyr, Lane 4—EcGyrA, Lane 5—MsGyrA, Lane 6—EcGyrB and Lane 7—MsGyrB; (**B**) EMSA with 72-bp DNA using MsGyrB and EcGyrB (0.1 and 0.2 µM) subunits. The DNA-bound complexes were resolved on a 5% native polyacrylamide gel; (**C**) Filter-binding assay with 20 nM each of MsGyrB, EcGyrB and *E. coli* ParE (EcParE) using 72-bp DNA. Enzyme was pre-incubated with 2 mM AMPPNP before addition of DNA (where indicated); (**D**) DNA (72 bp)-mediated stimulation of ATPase activity. ATPase assays were carried out with MsGyr (100 nM) or its GyrB subunit (100 nM) in the absence and presence of 72-bp DNA (400 nM). Lane 1—MsGyrB alone, Lane 2—MsGyrB + DNA, Lane 3—MsGyr alone, Lane 4—MsGyr + DNA and Lane 5—MsGyr + Novobiocin (10 µg/ml). The error bars represent standard deviation across three measurements.

To further analyse the distinct DNA-binding sites in *M. smegmatis* gyrase and determine to which subunit of the enzyme the second DNA molecule is bound, DNA crosslinking experiments were carried out using the holoenzymes and individual subunits. The holoenzymes, as well as both GyrAs, generated crosslinked species. Surprisingly, mycobacterial GyrB alone could crosslink to DNA while no crosslinked species was seen with *E. coli* GyrB (Figure 3A). To verify these results, EMSA was performed using the GyrB subunits from *E. coli* and *M. smegmatis.* Mycobacterial GyrB was found to bind DNA, while no detectable DNA-bound complex could be seen in the case of *E. coli* GyrB (Figure 3B). A more sensitive filter-binding assay was carried out to further assess the binding by GyrB subunits. In these assays, binding of both mycobacterial and *E. coli* GyrBs to DNA could be detected, although the lower degree of binding seen with *E. coli* GyrB would explain the inability to see the complex in EMSA (Figure 3C).

The two GyrB subunits dimerize in presence of ATP (33), forming the nucleotide operated (N) gate (34,46), with an internal cavity responsible for trapping the T-segment (35,47). This dimerization may influence the DNA-binding properties of GyrB. To test this, we carried out crosslinking experiments in the presence of ATP. The crosslinking of DNA to the *M. smegmatis* B subunit was reduced in presence of ATP (Supplementary Figure S3A). Binding of ATP and/or its hydrolysis thus leads to reduced interaction of GyrB subunit with DNA. To verify these results, 5′-adenylyl-β,γ-imidodiphosphate (AMPPNP), a non-hydrolysable analogue of ATP, was used in place of ATP in the crosslinking experiments. Formation of DNA–protein complex was reduced to a great extent when AMPPNP was incubated with GyrB before the addition of DNA (Figure 3C and Supplementary Figure S3B), indicating that the closure of the N gate induced by the non-hydrolysable analogue of ATP affected the DNA binding by GyrB.

GyrB subunits from different bacteria exhibit low level intrinsic ATPase activity, which is stimulated in the presence of DNA (48,49). ATPase assays were carried out in the absence and presence of duplex DNA using *M. smegmatis* DNA gyrase and its GyrB subunit alone to monitor whether DNA binding influenced the ATPase activity. The intrinsic ATPase activity of the subunit was stimulated >2-fold in the presence of DNA (Figure 3D). In contrast, ATPase activity of *E. coli* GyrB was not enhanced by DNA to a significant extent (data not shown), substantiating its poor interaction with the DNA shown in Figure 3C. *M. smegmatis* gyrase holoenzyme also showed a DNA-dependent increase in the ATPase activity, but the level of stimulation was almost equal to that found for GyrB alone, suggesting that the stimulation of the activity is because of DNA binding to GyrB. The ATPase activity was novobiocin-sensitive (Figure 3D), confirming that it was due to the GyrB subunit (50,51).

### Second DNA binding is an exclusive property of GyrB

**Figure 4.**
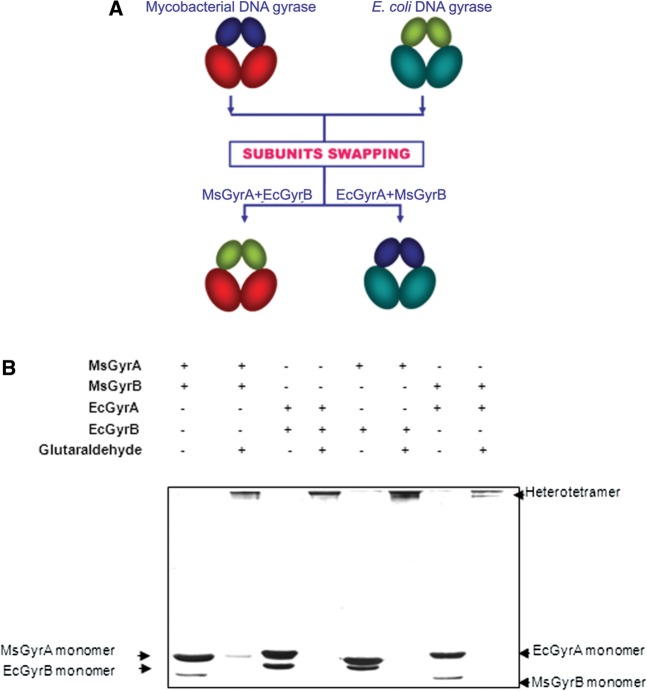
Swapping of subunits between MsGyr and EcGyr forms active heterotetramers. (**A**) Schematic representation of the experiment. MsGyrB—blue; MsGyrA—red; EcGyrB—green; EcGyrA—turquoise; (**B**) Glutaraldehyde crosslinking to show heterotetramer formation by EcGyrA + EcGyrB, MsGyrA + MsGyrB and MsGyrA + EcGyrB and EcGyrA + MsGyrB. The subunits were mixed and incubated on ice for 30 min. Crosslinking reaction with glutaraldehyde (final concentration—0.01%) was carried out at 37°C for 30 min. Native and crosslinked proteins were separated on 8% SDS–PAGE.

**Figure 5.**
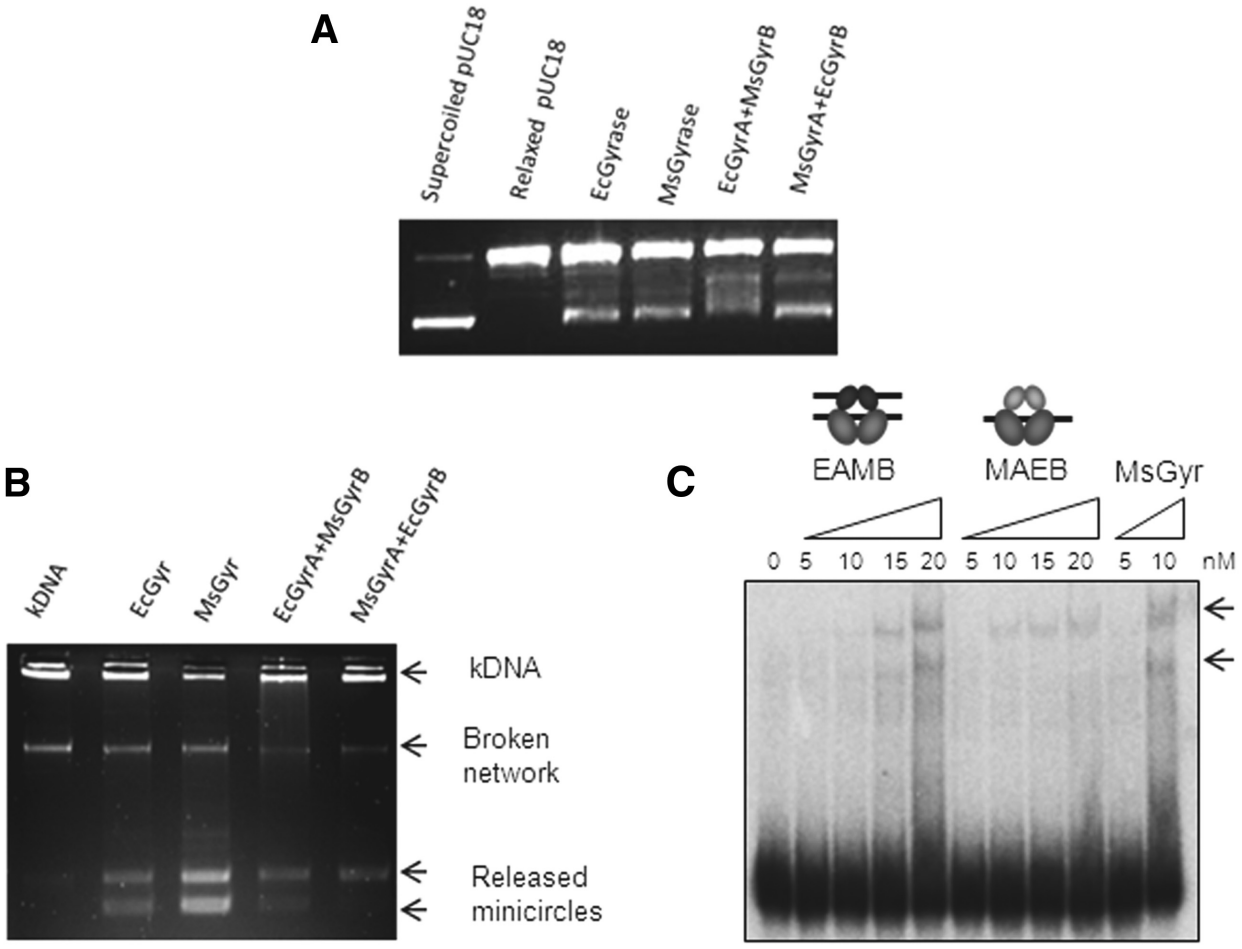
Topoisomerase activity of interspecies heterotetramers. (**A**) Supercoiling assay with the interspecies heterotetramers and the parent holoenzymes (25 nM). An amount of 300 ng relaxed pUC18 plasmid was used as substrate for the supercoiling assay. The topoisomers were resolved on a 1.2% agarose gel; (**B**) Decatenation activity of the parent and interspecies heterotetramers (25 nM) using 300 ng of kinetoplast DNA as substrate. The details of the reaction are given in ‘Materials and Methods’ section; (**C**) EMSA carried out with increasing concentrations (nM) of reconstituted hybrid heterotetramers (EAMB (EcGyrA + MsGyrB), MAEB (MsGyrA + EcGyrB) and MsGyr. 72-bp DNA was used as substrate. The DNA-bound complexes were resolved on a 5% native polyacrylamide gel. Arrows indicate the two complexes formed.

The above results strongly suggest a role for GyrB in capturing the second segment of DNA. However, to verify whether the GyrA or the holoenzyme take part in the process and to further establish the DNA-binding function of mycobacterial GyrB, interspecies heterotetrameric gyrases were reconstituted (Figure 4A). Crosslinking experiments carried out to verify heterotetramerization showed that *E. coli* GyrA can form a heterotetramer with *M. smegmatis* GyrB and similarly, *M. smegmatis* GyrA with *E. coli* GyrB. These crosslinked hybrids migrate similarly in the gel to their parent holoenzymes (Figure 4B). Heterotetramerization of the interspecies subunits was also verified by gel filtration; *M. smegmatis* GyrB was found to be co-eluting with *E. coli* GyrA and similarly *M. smegmatis* GyrA with *E. coli* GyrB (data not shown). The interspecies heterotetramers were catalytically active in the supercoiling reaction (Figure 5A). While the activity of the mycobacterial GyrA and *E. coli* GyrB hybrid was comparable to the parent holoenzymes, the heterotetramer containing the *E. coli* GyrA and mycobacterial GyrB showed reduced supercoiling activity (Figure 5A). However, importantly, this hybrid enzyme retained stronger decatenase activity (Figure 5B), a characteristic of mycobacterial gyrase (Figure 5B). *E. coli* DNA gyrase, as expected showed lower decatenation activity (39). The interspecies hybrids were analysed for their DNA-binding characteristics by EMSA. The re-constituted heterotetramers bound DNA, and importantly, *E. coli* GyrA and *M. smegmatis* GyrB complex could bind to two DNA molecules, similar to *M. smegmatis* DNA gyrase holoenzyme. The other mixed heterotetramer having *M. smegmatis* GyrA and *E. coli* GyrB was found to bind only one DNA, like *E. coli* DNA gyrase holoenzyme (Figure 5C). From these observations, we deduce that the binding to the second DNA molecule is an intrinsic property of GyrB subunit and GyrA subunit or the holoenzyme does not appear to influence the process in a significant fashion.

### DNA binding by ParE and stimulation of ATPase activity

**Figure 6.**
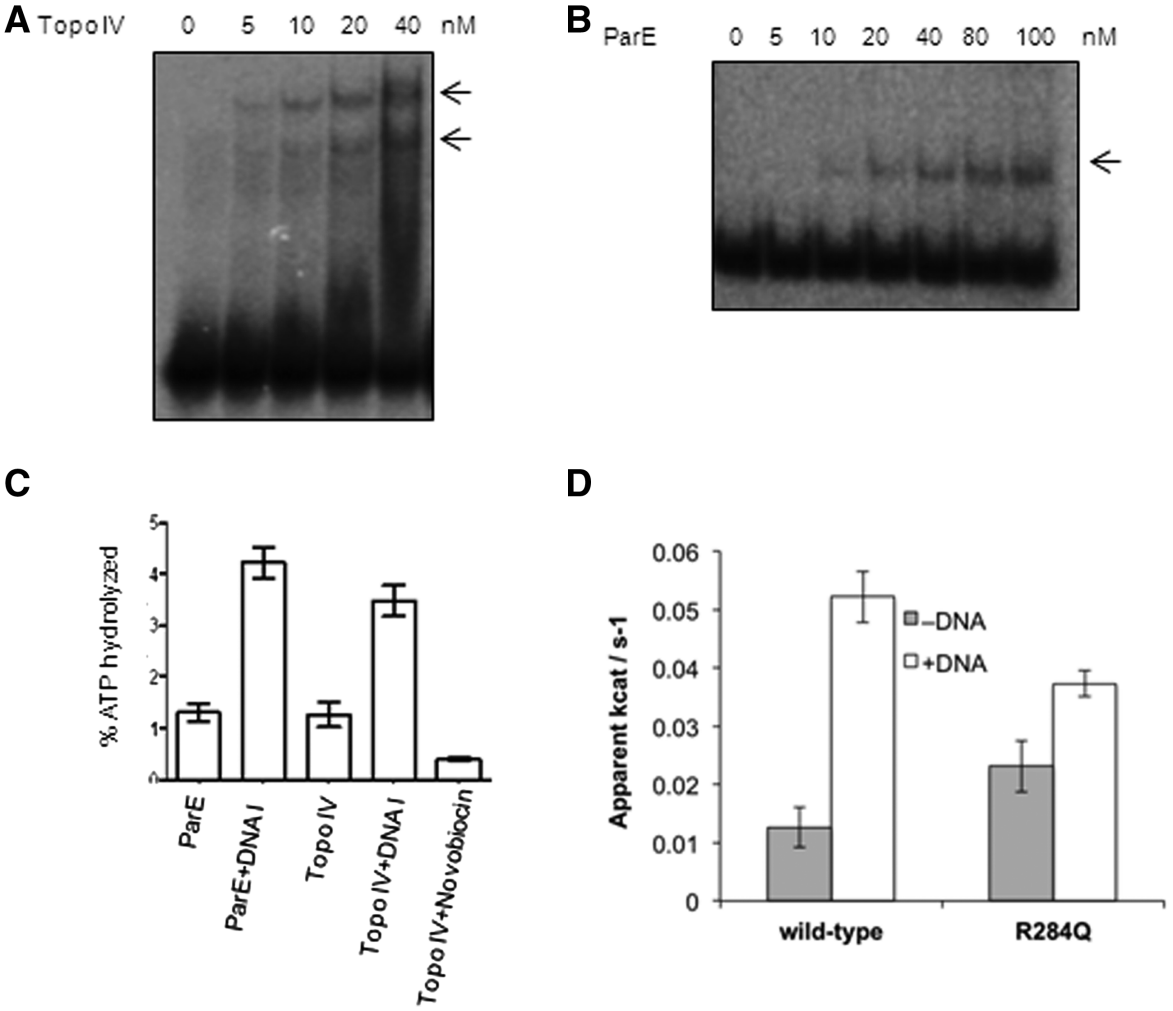
DNA binding and ATPase activity of *E. coli* topoIV and its ParE subunit (**A**) EMSA with *E. coli* topo IV holoenzyme (5–40 nM) using 72-bp DNA. The arrows indicate two DNA-bound complexes; (**B**) EMSA with increasing concentrations of ParE (5–100 nM) subunit using 72-bp DNA. The reaction conditions were similar to those used for Figure 1; (**C**) ATP hydrolysis by the topo IV holoenzyme and ParE (100 nM of each) subunit in the absence and presence of 72-bp (100 nM) DNA; (**D**) ATPase activity of wild-type and R284Q mutant ParE43 fragment (2 μM dimer), in the presence and absence of linear pBR322 at a ratio of 25-bp DNA per protein dimer. The activity is expressed as the apparent *k*_cat_ of the protein dimer in the presence of 2 mM ATP. The error bars represent the standard deviation of three measurements.

The ability of mycobacterial DNA gyrase to capture two separate DNA molecules may enable it to act as a strong decatenase, by allowing it to capture a T-segment in *trans*. To further test this proposition, we investigated the binding of DNA to *E. coli* topo IV, one of the strongest known decatenases (13,19,52), using the same experimental strategy. EMSA with *E. coli* topo IV showed the formation of two bound complexes (Figure 6A). Biotin pulldown experiments with topo IV (Supplementary Figure S4, similar to the experiments described for gyrase in Supplementary Figure S2) confirmed the above finding. Next, to check whether this property of binding to second DNA is conferred by ParE (the homologue of GyrB in topo IV), we carried out EMSA with the 72-bp DNA. ParE subunit alone was found to be able to bind DNA (Figure 6B). Filter-binding assays showed that DNA binding by ParE was most efficient among the proteins tested (Figure 3C). Furthermore, the intrinsic low-level ATPase activity of ParE was enhanced >3-fold by the addition of DNA (Figure 6C). In a separate set of experiments, ParE43 ATPase domain (the amino terminal 43-kDa fragment of ParE, Supplementary Figure S5) bound DNA and the binding is greatly reduced in presence of non-hydrolysable ATP analogue (Supplementary Figure S5), similar to the behaviour of mycobacterial GyrB (Supplementary Figure S3B). The ParE43 ATPase domain showed 4-fold stimulation of ATPase activity in the presence of linear DNA (Figure 6D). Topo IV holoenzyme also showed DNA-dependent stimulation of ATPase activity (Figure 6C). However, the stimulation appears to be ParE-specific as in the case of GyrB from *M. smegmatis* (Figure 3D); the increased activity of the holoenzyme was to the same extent as that of ParE, indicating that ParE is mainly responsible for binding to the second DNA fragment.

### A conserved arginine residue within the ATPase domain is required for efficient second DNA binding

**Figure 7.**
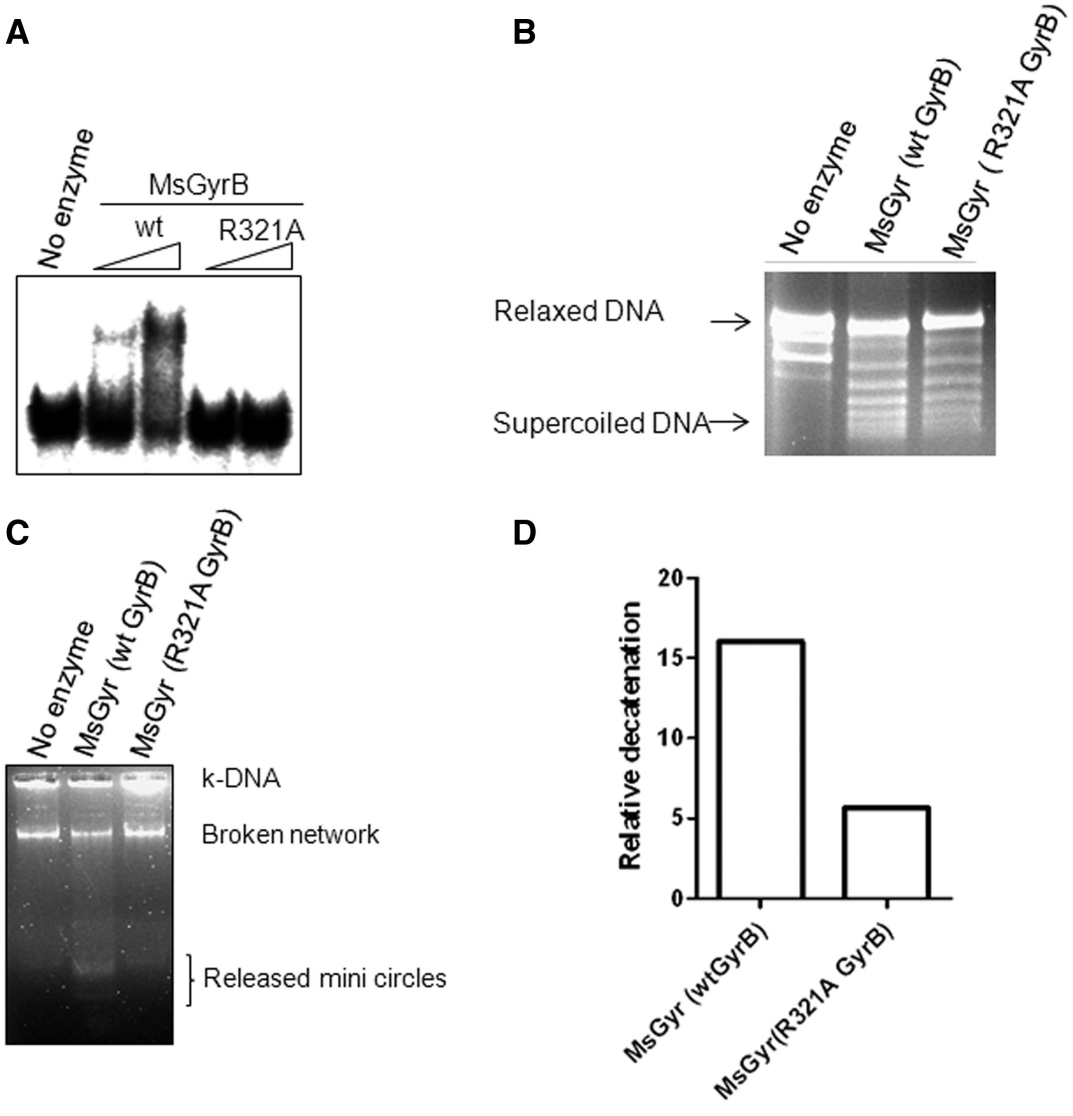
DNA-binding activity of the wild-type (wt) and R321A GyrB subunit. (**A**) EMSA with 200 and 400 nM each of wt MsGyrB and its R321A mutant. The reaction conditions were similar to those used for Figure 2B. The 72-bp DNA was used as binding substrate; (**B**) DNA supercoiling of the mycobacterial gyrase reconstituted by mixing the wt or the R321A GyrB with GyrA subunit. An amount of 25 nM of *M. smegmatis* gyrase was reconstituted by mixing 25 nM of GyrA with 50 nM of either wt or R321A GyrB. The details of the reaction are given in ‘Materials and Methods’ section; (**C**) Decatenation assay with the holoenzyme reconstituted by mixing 25 nM of GyrA with 50 nM of either wt or R321A GyrB. An amount of 300 ng of kDNA was used as substrate. The decatenated minicircles were resolved on a 1.2% agarose gel; (**D**) Quantitation of the decatenation (released minicircles) by the wt and R321A GyrB containing holoenzyme from *M. smegmatis* (in Figure 7C) using Fujifilm multigauge V2.3.

The crystal structure of the 43-kDa GyrB (35) and ParE (32) subunits from *E. coli* revealed an arginine residue in the cavity of the ATPase domain that is found to be conserved in other type IIA topos and which may have a role in DNA binding. The side chain of this arginine protrudes into the cavity formed by the ParE dimer. This arginine is also found in *M. smegmatis* GyrB (R321), although the protein shows only 40% identity to *E. coli* GyrB. The mutation of this conserved arginine to alanine in *M. smegmatis* GyrB (R321A) significantly reduced the DNA-binding ability of the mutant. As a result, a DNA–protein complex is not seen in EMSA (Figure 7A). A filter-binding assay also showed reduction in the binding ability of the R321A GyrB (data not shown). Importantly, the holoenzyme reconstituted with R321A GyrB was unperturbed in supercoiling activity (Figure 7B) but found to be compromised in its decatenation ability (Figure 7C and D). The reduced DNA-binding efficiency of the R321A also resulted in the diminished DNA-dependent stimulation of the ATPase activity of the individual subunit or the reconstituted holoenzyme (Supplementary Figure S6A and B). This is consistent with the effect of an R284Q mutation, the homologous position in *E. coli* ParE in reducing the DNA binding (Supplementary Figure S5) and stimulation of ATPase activity of the ParE43 from 4-fold in the wild-type to 1.6-fold in the mutant (Figure 6D).

## DISCUSSION

In the reaction mechanism proposed for DNA gyrase, the CTD of GyrA is involved in wrapping the DNA around the holoenzyme, allowing a T-segment from the same molecule to enter the GyrB clamp (53). While this mechanism explains the intra-molecular strand passage required for gyrase supercoiling, it is not clear how topo IV and other non-supercoiling type II enzymes, which use a T-segment from another DNA molecule during decatenation, capture DNA into the ParE / ATPase subunit clamp. We have demonstrated that both *M. smegmatis* DNA gyrase and *E. coli *topo IV, but not *E. coli* gyrase, can bind to two DNA fragments; this ability is thus correlated with significant decatenation activity. The first complex appears to be due to the binding of G-segment DNA, as judged by the stabilization of this complex by MFX. Experiments with isolated GyrB/ParE subunits, and subunit swapping suggest that the second DNA binding is conferred by GyrB/ParE. The reduction in this binding caused by pre-closure of the clamp with AMPPNP, or by substitution of a prominent arginine residue on the inner surface of the GyrB/ParE cavity in both *M. smegmatis* GyrB and *E. coli* ParE strongly suggest that the second DNA binds in the GyrB/ParE cavity, forming a T-segment.

Different type II topos have preference for a particular geometry of the substrate recognized by their respective CTD of the cleavage religation subunit (7,25,27,28,54). The CTD of GyrA wraps the DNA in *cis* to bring it inside the GyrB clamp as a T-segment (53). Removal of the CTD of *E. coli* GyrA converts it into a conventional non-supercoiling type IIA topo (55), which can relax supercoils in the presence of ATP. This C-terminal truncation enhances decatenation activity of *E. coli* gyrase suggesting that the CTD may exert a negative effect on decatenation activity. Additionally, the CTD of ParC and GyrA show remarkable sequence and structural differences (24,25). The presence of a β-strand bearing proline in the GyrA CTD introduces an out of plane twist in the β-pinwheel domain, causing an abrupt bend in the DNA-binding surface, thus contributing to the unique negative supercoiling activity of DNA gyrase (56). ParC CTD, on the other hand has been shown to contain a broken β-pinwheel domain different from that of GyrA CTD, resulting in lower DNA bending (27). These structural studies indicated that GyrA/ParC CTD curvature may determine the enzyme’s ability to present the T-segment (25,27,55,56). Our present studies suggest that an additional DNA-binding site in the ATPase cavity is required for efficient T-segment capture in a decatenation reaction.

The ATPase domain of human Topo IIα has also been shown to bind DNA, resulting in stimulation of its ATPase activity (57,58). *Plasmodium falciparum* GyrB subunit which is selectively localized to a specialized organelle termed the apicoplast, has been shown to bind DNA and the binding stimulates its ATPase activity (59). A point to be noted is that, in the apicoplast genome, decatenation would be a prerequisite when the organelle divides along with the parasite after the replication process. These studies also point towards the importance of the ATPase subunit in capturing the T-segment. The interaction of Drosophila type II topo at DNA crossovers has also suggested the binding to an additional DNA-binding site (60). All these observations suggest the need for efficient capture of a second DNA in a decatenation reaction. However, the exact mechanism by which ATPase domain captures DNA during the process remains to be understood.

From the analysis of GyrB/ParE sequences, it is rather difficult to predict which of the residues contribute to DNA binding. Distribution of the basic amino acid patches over a large surface of GyrB/ParE renders such analysis a daunting task. Although high-resolution structures of intact GyrB/ParE are not available, the structural analysis of the ATPase domain of GyrB and ParE subunit of *E. coli* showed an arginine from each monomer protruding into the cavity (32,35). Mutation of this residue (R284Q) in the ATPase domain of *E. coli* ParE (ParE43) resulted in decreased stimulation of ATPase activity (Figure 6D) and reduced DNA binding (Supplementary Figure S5), similar to results with the homologous mycobacterial GyrB mutant described in this manuscript (Figure 7A and Supplementary Figure S6). Notably, the holoenzyme comprising the R321A GyrB retained its supercoiling activity (Figure 7B) but the decatenation activity was affected (Figure 7C and D). In contrast, the mutation in the conserved arginine in *E. coli* GyrB (R286Q) led to the loss of both supercoiling and decatenation activities (47). Taken together, these results suggest that the arginine has an important role in T-segment interaction. However, the presence of this arginine is not a crucial determinant of decatenation activity, since the residue is conserved in many other ‘normal’ gyrase sequences in addition to *E. coli* GyrB; rather these results point to the importance of efficient capture of a T-segment in the GyrB cavity for decatenation activity. Since the mutation of the residue in the two gyrases show somewhat different properties, it is likely that additional residues are involved in the efficient binding of the T-segment in case of mycobacterial GyrB and *E. coli* ParE.

While the supercoiling activities of DNA gyrase from *E. coli* and *M. smegmatis* are comparable, they differ significantly in their decatenation ability (39). Based on our present findings, we predict a strong decatenation activity for DNA gyrase from diverse bacteria that lack topo IV. In all eubacteria containing a single type II topo, DNA gyrase may have the task of catalysing both the supercoiling and decatenation reactions. An interesting question is whether and how the enzyme is able to switch from a supercoiling to a decatenation mode at the end of DNA replication and other intracellular situations that require untangling of DNA. In our experiments, we have demonstrated that altering substrate DNA length can reveal the additional DNA-binding site in mycobacterial GyrB or *E. coli* ParE. With a long DNA substrate (240 bp), a single binding event is observed indicating that GyrA CTD-mediated writhing step facilitates intra-strand capture, thus favouring the supercoiling reaction. Indeed electron microscopic studies carried out earlier with *M. luteus* gyrase demonstrated looped structures indicating the intra-strand capture of the T-segment with longer DNA molecules (61). When shorter DNA fragments were used in the reaction, the mycobacterial gyrase was able to capture two distinct DNA molecules, a prerequisite for decatenation. When a very small-length DNA (40 bp) was used, it formed only one bound complex (Figure 1A), perhaps because of the inability of the GyrB to bind smaller DNA (data not shown). Earlier studies have suggested that DNA <50 bp can be used as a G-segment which is not effective as T-segment (62). However, gyrase would be present on a long DNA molecule *in vivo* and hence, this will not suffice to explain the distinction between two styles of reaction. One possibility is that under some conditions, perhaps when negative supercoiling is high, the torsion in the DNA opposing the positive wrap would allow an inter-molecular T-segment to compete more easily with the wrapped T-segment. Topo-interacting proteins could also play a major role in this switch *in vivo*, as demonstrated in case of topo IV of *E. coli* (63).

To conclude, efficient capture of the transfer segment by the ATPase subunit of type IIA topos is a key event during the decatenation process. While the CTD of the DNA breakage/religation subunit (GyrA/ParC) has determinants for recognition of the appropriate substrate for strand cleavage and discrimination between DNA of different topologies (24,25), capture of the T-segment seems to be an exclusive property of the ATPase subunit of the strong decatenases. In principle, inhibition of the binding of the second DNA to the enzyme would inhibit the overall reaction of the topos. The present studies hence open up the possibility of new strategies for the design of novel classes of gyrase inhibitors against the mycobacterial enzyme and possibly for other strong decatenases.

## SUPPLEMENTARY DATA

Supplementary Data are available at NAR Online: Supplementary Table 1 and Supplementary Figures 1–6.

## FUNDING

Senior research fellowship from Department of Biotechnology, Government of India (to R.K.); BBSRC research studentship (to J.E.R.); Wellcome Trust grant [GR058618 to A.D.B.]; J. C. Bose fellowship of Department of Science and Technology and Centre for Excellence grant from Department of Biotechnology, Government of India (to V.N.). Funding for open access charge: J. C. Bose fellowship of Department of Science and Technology.

*Conflict of interest statement*. None declared.

## Supplementary Material

Supplementary Data
