# Supplementary material for: Binding of two DNA molecules by type II topoisomerases for decatenation
Source: Nucleic Acids Res. 2012 Sep 18;40(21):10904–15. doi: 10.1093/nar/gks843 (PMC3510509; doi:10.1093/nar/gks843)
Supplement: Supplementary Data [file supp_40_21_10904__index.html]

Binding of two DNA molecules by type II topoisomerases for decatenation — Binding of two DNA molecules by type II topoisomerases for decatenation — Supplementary Data 

# Binding of two DNA molecules by type II topoisomerases for decatenation

## Supplementary Data

files

**Files in this Data Supplement:**

- Supplementary Data - pdf file
