# Supplementary material for: Binding of two DNA molecules by type II topoisomerases for decatenation
Source: Nucleic Acids Res. 2012 Sep 18;40(21):10904–15. doi: 10.1093/nar/gks843 (PMC3510509; doi:10.1093/nar/gks843)
Supplement: Supplementary Data [file supp_gks843_nar-01280-f-2012-File009.pdf]

**DNA I (40 bp)**	5’-GGT TGG ACT AAG TCC CCA GGC CCC TTA GCC ACC ACA ACG T-3’
**DNA II (72 bp)**	5’-ACT CTA GAG GAT CCC CGG TTG GAC TAA GTC CCC AGG CCC CTT AGC CAC CAC AAC GTG GGT ACC GAG CTC GAA-3’
**DNA III (143 bp)**	5’-GTA AAA CGA CGG CCA GTG CCA AGC TTG CAT GCC TGC AGG TCG ACT CTA GAG GAT CCC CGG TTG GAC TAA GTC CCC AGG CCC CTT AGC CAC CAC AAC GTG GGT ACC GAG CTC GAA TTC GTA ATC ATG GTC ATA GCT GTT TCC TG-3’
**R321Afor**	5′ - TAA TAC GAC TCA CTA TAG GG - 3′
**R321Arev**	5’ - GGT CAG CGC CGC GGC GAA ACC CTC C – 3’

Supplementary Table 1. Sequences of DNA molecules used in this study (top strand).

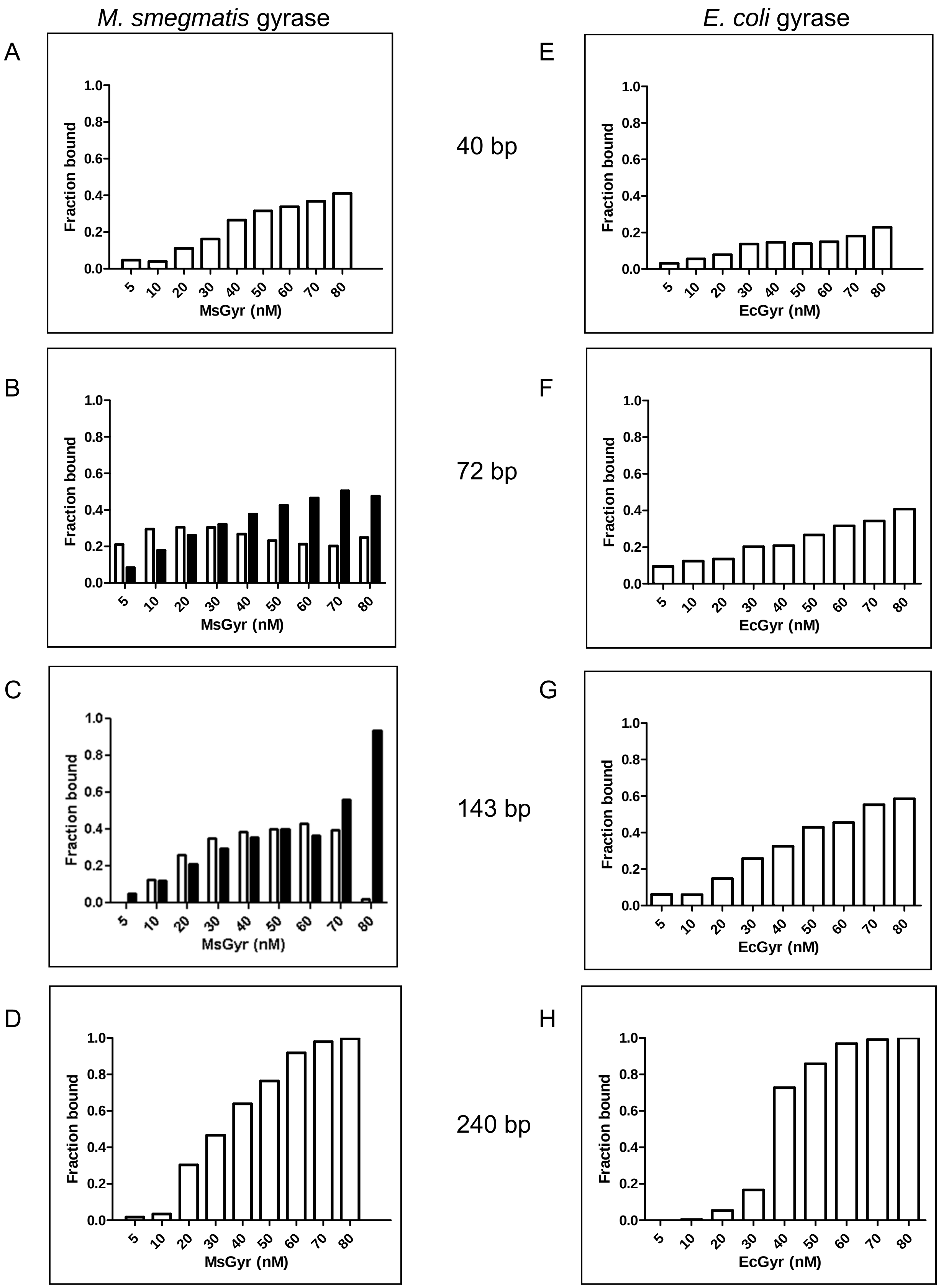

Supplementary Figure S1. Densitometric quantification of the DNA bound complexes in the gels presented in Figure 1. Each panel corresponds to its respective panel in Figure 1. The DNA bound complexes were quantified using Multi Gauge V2.3 software. The fraction of the DNA bound was plotted against each enzyme concentration. Panels B and C show the quantification of the two complexes formed in Figure 1 B and C (Faster moving complex es are represented as hollow bars and the slower moving complexes as solid bars in these two panels).

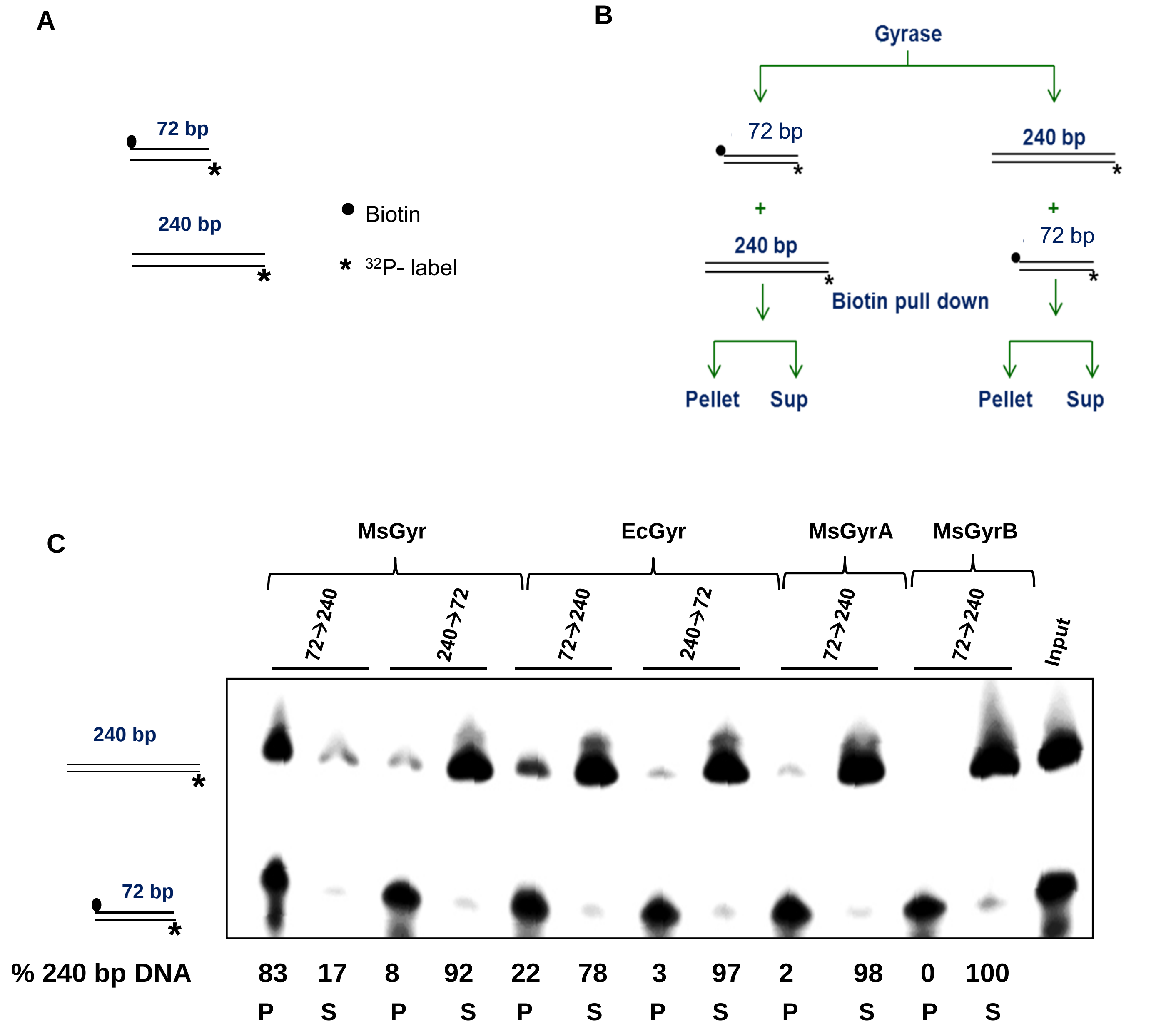

Supplementary Figure S2. Biotin pulldown assay. **(A)** The DNA molecules used in the assay. The 72 bp DNA was biotinylated and ^{32}P-labeled; 240 bp DNA was ^{32}P-labeled and not biotinylated; **(B)** Schematic of the biotin pulldown assay; **(C)** 200 nM each of DNA gyrase from *M. smegmatis* and *E. coli* and mycobacterial gyrase subunits were used. The DNA molecules were added in the order as shown in the schematic. Reactions were performed at 25˚C. The percentage fraction of 240 bp DNA in pellet and supernatant (sup) are indicated. P- pellet and S- sup. Details of the reaction carried out are given in Materials and Methods.

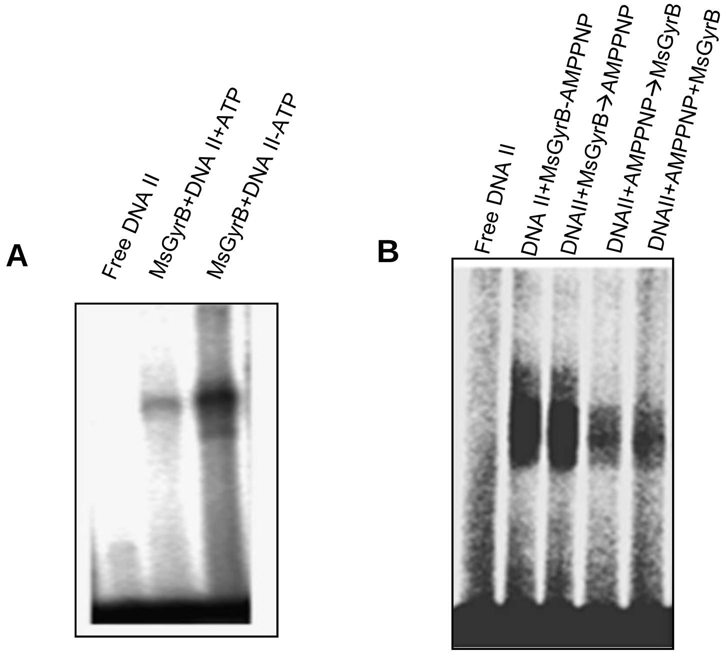

Supplementary Figure S3. (A) Crosslinking carried out with MsGyrB (1 μM) and 72 bp DNA in the absence and presence of ATP (2mM) as indicated; **(B)** DNA crosslinking in the presence of AMPPNP (2mM) with different orders of addition. Lane 1- DNA alone, lane 2- MsGyrB + DNA , lane 3- MsGyrB + DNA followed by the addition of AMPPNP, lane 4 – MsGyrB + AMPPNP followed by the addition of DNA, lane 5 – Both AMPPNP and DNA are added together to MsGyrB. DNA crosslinking reactions were carried out by addition of cross-linking solution (37% formaldehyde and Methanol; 9:1) to a final concentration of 0.7%. The crosslinked products were resolved on 6 % SDS polyacrylamide gels.

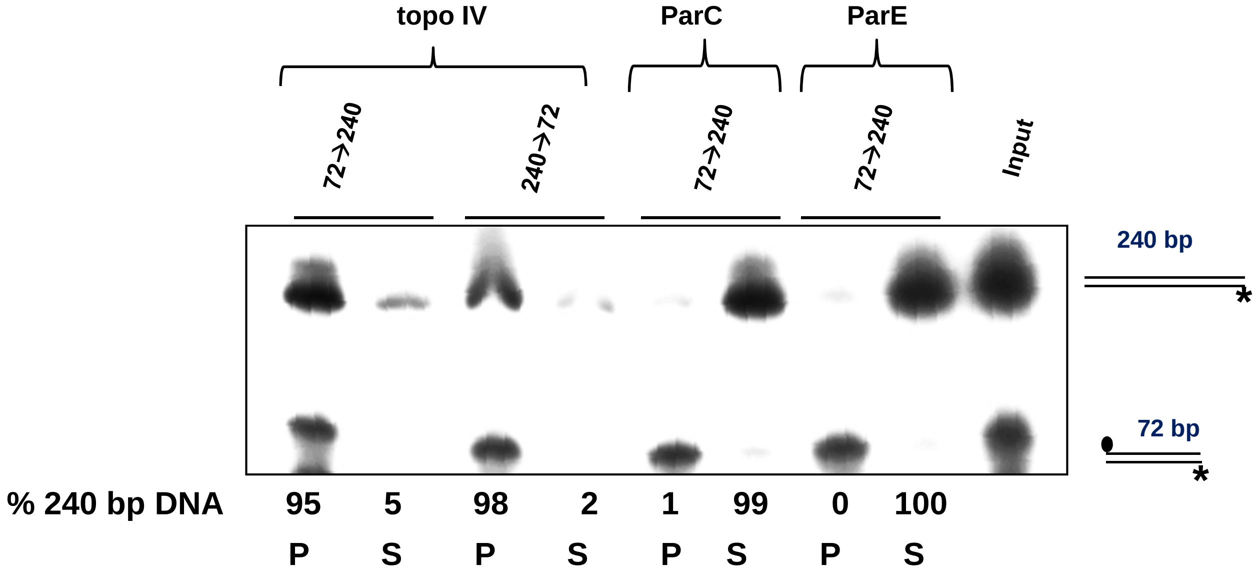

Supplementary Figure S4. Biotin pulldown assay with topo IV and its individual subunits (each 200 nM). Biotinylated and radiolabelled 75 bp DNA and radiolabeled 240 bp DNA were incubated with 200 nM of topo IV at 25˚C for 10 min. Streptavidin beads were used to pull down the biotinylated DNA (see Materials and Methods). Percentage fraction of 240 bp DNA in pellet was quantified using Multi Gauge V2.3 software.

A

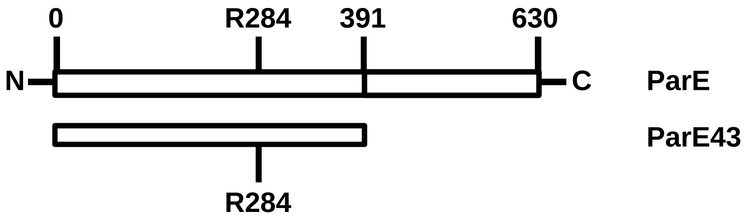

B

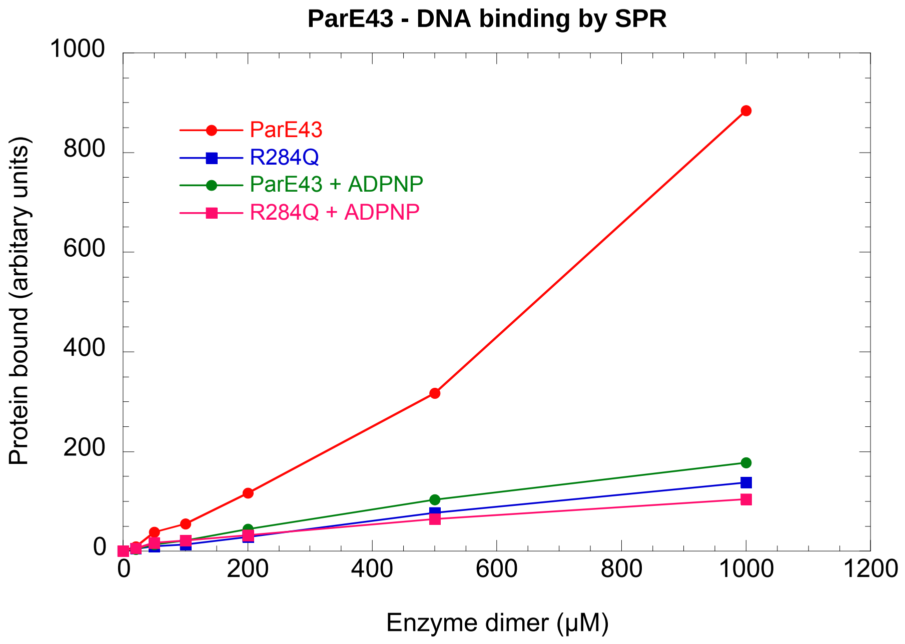

Supplementary Figure S5. (A) Schematic representation of the full length ParE and its N terminal domain (ParE43) from *E. coli*. The conserved arginine at position 284 is shown; **(B)** DNA binding to a 140 bp DNA fragment by wild type and R284Q mutant of ParE43 ATPase domain of *E. coli* topo IV in presence and absence of ADPNP, measured by SPR. The protein bound to the DNA is plotted against enzyme dimer concentration (μM). Both the mutation of the most prominent arginine residue in the protein cavity, and pre-incubation with the non-hydrolyzable ATP analogue, ADPNP, which will form the closed dimer conformation of the protein, lead to a significant reduction in the DNA binding ability of the ATPase domain.

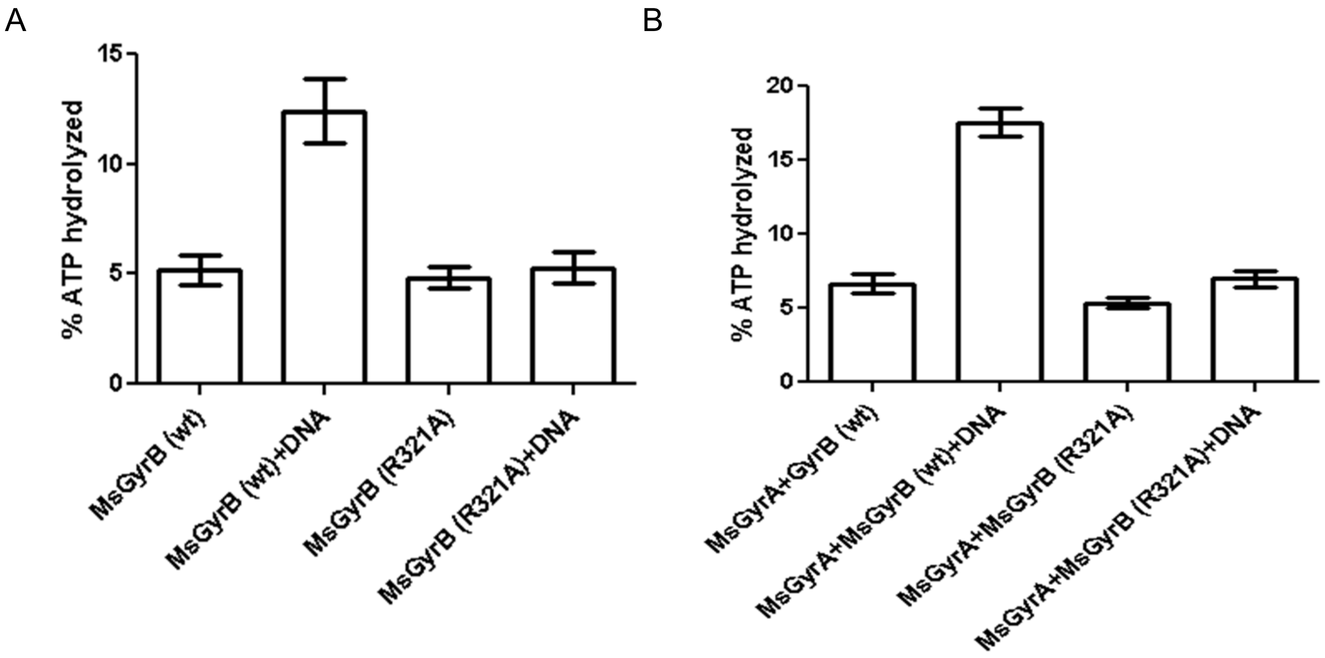

Supplementary Figure S6. Intrinsic and DNA-dependent stimulation of the ATPase activity of **(A)** the wild type and R321A mutant of *M. smegmatis* GyrB; and **(B)** the gyrase holoenzyme reconstituted with wild type or R321A mutant of mycobacterial GyrB. 100 nM of each of the subunit or holoenzyme was used in the assay. For stimulation of the ATPase activity 400 nM of 72 bp DNA was used.
